# Efficacy of an Internet-Based Intervention for Subclinical Depression (MoodBox) in China: Study Protocol for a Randomized Controlled Trial

**DOI:** 10.3389/fpsyt.2020.585920

**Published:** 2021-01-12

**Authors:** Xu Chen, Xiaolong Zhang, Xuequan Zhu, Gang Wang

**Affiliations:** ^1^Beijing Key Laboratory of Mental Disorders, The National Clinical Research Center for Mental Disorders, Beijing Anding Hospital, Capital Medical University, Beijing, China; ^2^Advanced Innovation Center for Human Brain Protection, Capital Medical University, Beijing, China

**Keywords:** subclinical depression, internet-based, digital intervention, psychotherapy, prevention, RCT - randomized controlled trial

## Abstract

**Background:** Subclinical depression is a prevalent mental health problem and increases the incidence of the onset of major mood disorders, such as major depressive disorder (MDD). Psychological interventions have been proved to be effective for reducing depressive symptoms for people with subclinical depression and can prevent the onset of MDD. However, people have limited access to face-to-face psychotherapy. Internet-based psychological intervention is an alternative treatment option. The aim of the study is to evaluate the efficacy of MoodBox, an online psychological intervention program, for subclinical depression.

**Methods:** This study is a multicenter, randomized, controlled, non-blinded superiority study with three parallel groups. A total of 435 first-year university students with subclinical depression will be recruited. Eligible participants will be randomly assigned to the MoodBox group, the online psychoeducation group, and the naturalistic observation group at a ratio of 1:1:1. The intervention period is 8 weeks, and participants will be continuously followed up for 1 year. The primary outcome of the study is the efficacy of the intervention, defined as measured by the Patient Health Questionnaire (PHQ-9).

**Discussion:** This is the first study to innovatively develop and test an intervention to improve psychological well-being and decrease the incidence of MDD in a subclinical depression population in China. Once proven effective and acceptable, MoodBox could be potentially integrated into the routine clinical service to facilitate the management for people with subclinical depression.

**Clinical Trial Registration:** The trial is registered with the Chinese Clinical Trial Registry on 21 July 2020 (No. ChiCTR2000034826).

## Introduction

Subclinical depression, also known as subthreshold depression, subsyndromal depression, or minor depression, is considered to be a condition that does not meet the diagnostic criteria for depression but present with at least two and no more than four depressive symptoms, which must include one of the core symptoms of major depressive disorder (MDD) (i.e., depressed mood or anhedonia) and last a duration for at least 2 weeks (1–3). The prevalence of subclinical depression ranges from 1.3 to 17% in primary care and from 1.4 to 17.2% in community settings (2). Subclinical depression has detrimental effects on psychosocial function and quality of life, increases service utilization, and carries a high risk of developing into a full-blown depression (4, 5). Furthermore, a meta-analysis indicated that the mortality rates in subclinical depression are comparable with those in MDD (6).

Although depressive disorders are the most frequent consequence of subclinical depression, it can also lead to other mood disorders. From the clinical staging approach, subclinical depression could be the prodromal stage for a variety of mental health problems (7). However, studies on subclinical depression mostly focus on preventing the onset of depression, and other mood disorders are commonly overlooked (8, 9). Moreover, in light that depressive episode is usually the first episode of bipolar disorder (10), especially in young age, the reduction of depressive symptoms is not equivalent to remission of subclinical depression; alternatively, it may be a sign for the development of mania or hypomania. Therefore, a comprehensive assessment of mood events is necessary for subclinical depression.

Psychotherapy is effective in reducing depressive symptoms for people with subclinical depression and can prevent the onset of MDD (11). Although medication is recommended to treat depression, no evidence has supported the use of medication for subclinical depression (12). Cognitive behavioral therapy (CBT), interpersonal psychotherapy (IPT), problem-solving therapy (PST), and internet-based psychotherapy are primary choices for treating subclinical depression and preventing depression (13). However, traditional psychotherapy (i.e., face-to-face therapy carried out by a therapist) is not suitable for the preventive use in a large population, due to the limited number of qualified therapists and high costs (14–16). Moreover, in China, currently, mental health service is prioritized for people who meet the clinical threshold, and people with subclinical conditions are largely underserved (17).

Internet-based psychotherapy has the advantage of being accessible at any time and place, people can work in their own pace, and at-risk individuals can be reached more timely as compared with traditional face-to-face approach (18). Therefore, an effective internet-based psychological intervention has the potential to meet the need for the underserved population. Although internet-based interventions have been proved effective in western countries (18–20), to date, in China, no internet-based psychological intervention for subclinical depression has been developed and tested. To address the gap, informed by evidence-based psychological interventions for subclinical depression (e.g., CBT and IPT), we developed an internet-based psychological intervention program, named MoodBox, for people with subclinical depression, and we carried out a randomized controlled trial (RCT) to test the efficacy of the program. The primary aim of the study is to evaluate the efficacy of MoodBox for subclinical depression. The secondary aims are to evaluate the feasibility, usability, acceptability, and safety of MoodBox. To the authors' knowledge, MoodBox is the first internet-based intervention for subclinical depression in China.

## Methods

### Study Design

**Figure 1:**
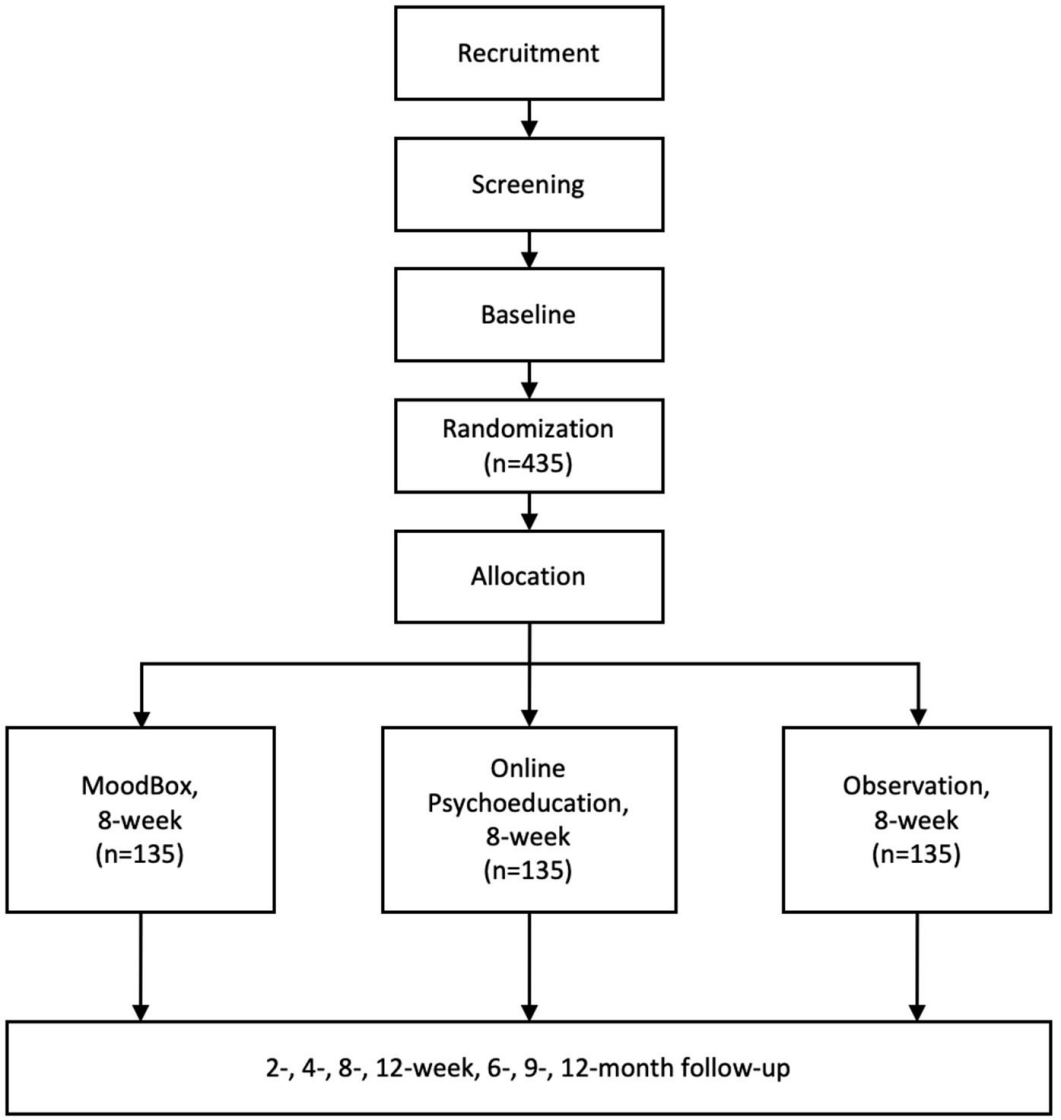
Overview of the study design.

The study is a multicenter, randomized, controlled, non-blinded superiority study with three parallel groups. People with subclinical depression will be invited to participate in the study. Eligible participants will be randomly assigned to one of the three groups—the MoodBox group, the online psychoeducation group, and the naturalistic observation group—at a ratio of 1:1:1. By designing a three-arm study and choosing online psychoeducation and naturalistic observation as comparators, we aim to minimize the influence of digital placebo effect and the natural remission of subclinical depression. The intervention period of the study is 8 weeks. Following the completion of the intervention period, the participants will be continuously followed up for 1 year. All assessments will be conducted remotely using a social media app (WeChat) to send out notifications to participants. At each follow-up time window, a notification will be sent out via the WeChat public account to remind the participant to fill out a set of self-report scales. The participants will be asked to report general information, symptom severity, social functioning, childhood traumatic experiences, parenting experiences, attachment, and coping style at the baseline and to report their symptom severity and social functioning at each follow-up time point. After the baseline assessment, the participants will be assessed at 2, 4, and 6 weeks after they started to receive the intervention and a post-intervention assessment at 8 weeks. After that, participants will enter the follow-up phase and will be assessed at 12-week, 6-month, 9-month, and 12-month follow-up time points. See Figure 1 for a flowchart demonstrating the study procedure in detail. The study has been registered as a clinical trial on the Chinese Clinical Registry website (Registration number: ChiCTR2000034826).

### Participation Eligibility

The eligibility of participants will be assessed in accordance with the following inclusion and exclusion criteria by psychiatrists through clinical interview and relative scales. The inclusion criteria are (1) age 18 years or older, no restriction for gender; (2) the score of two to four items on the Patient Health Questionnaire (PHQ-9) scale ≥ 2 (must include the first or second item, which are the indicator for core symptoms of depression) and a total score ≤ 9; (3) not meeting diagnostic criteria for any mental disorders according to the *Diagnostic and Statistical Manual of Mental Disorders* (Fourth Edition) (DSM-IV); and (4) being able to give consent. The exclusion criteria are (1) having severe and unstable physical illness; (2) having organic brain damage; or (3) being alcohol or drug dependent. A psychiatrist will be using Mini-International Neuropsychiatric Interview (MINI) to rule out any mental disorders. The exclusion criteria will be assessed through clinical interview by completing a screening checklist of the study.

### Sample Size

Four hundred thirty-five participants are intended to enroll and will be randomized with a ratio of 1:1:1. The estimated sample size provides 80% power (two sided α = 0.025, considering a multiple comparison) to detect difference in the primary outcome and the proportion of participants without subclinical depression between the MoodBox group with the online psychoeducation group and the observation group in a survival analysis (assuming the minimum difference is 14%) after 8 weeks. Taking into account a predicted 20% attrition for this comparison, at least 145 participants would be needed in each group. The calculation was based on a meta-analysis that reported clinical difference between the internet-based intervention group and psychoeducation group (9), and the calculating methods were in accordance with Chow et al.'s instruction (21).

### Recruitment and Randomization

The study is a multicenter, three-arm, parallel RCT. The study will be conducted at three universities in Beijing, China. First-year students who are waiting for the regular physical examination will be invited by research staff to complete a brief screening questionnaire to assess their eligibility. Students who meet the eligibility criteria will be provided with the participant information sheet and the consent form of the study. If the participants were interested in participating in the study, research staff would give a more comprehensive face-to-face explanation of the study in a private consultation room. Written consent will be required before the participant formally enrolls in the study. Eligible participants will be randomly assigned to three groups—the MoodBox group, the online psychoeducation group, or the observation group—with a ratio of 1:1:1. Randomization will take place after the completion of baseline assessment using a randomization code, which will be developed in SAS 9.4 (SAS Institute Inc., Cary, NC, USA) by a statistician who is not involved in the study.

### Intervention

#### MoodBox Group

The MoodBox group will receive an 8-week internet-based psychotherapy program. The program is a web-based psychological intervention informed by evidence-based psychological interventions, including CBT, IPT, and mindfulness meditation. The program consists of four core modules, which are symptom assessment, cognitive therapy, interpersonal therapy, and relaxation. There are 20 sessions in total, and each session takes approximately 30 min to complete. Participants are expected to complete a minimum of two sessions per week. After finishing each session, participants will receive a virtual trophy in the program as a reward. Participants will be assigned with a user account and receive training of the program at the first visit.

An outline of a brief description of the internet-based intervention program contents can be found in Table 1. The first module (session 1) of MoodBox is an introduction of the program that provides a comprehensive explanation of depression and the program, and there is an assessment of depression using PHQ-9 at the end of this session. The second module is cognitive intervention (sessions 2–11), which is based on cognitive reconstruction technique of CBT. This module will illustrate by animation the different types of automatic thoughts that are related to depression. After the illustration, participants will be required to identify their own automatic thoughts and to practice modifying the automatic thoughts by doing exercises in the program. The third module is interpersonal intervention (sessions 12–18). This module is based on IPT theory and focuses on interpersonal problems related to depression. The fourth module is relaxation, which includes mindfulness meditation and relaxation music. This module is not stand-alone; instead, it was embedded in other modules and would be presented after finishing each session. The final module is review and termination (sessions 19 and 20). In this module, participants will review all the previous sessions and will be given advice for their future life.

**Table 1:** The content of the MoodBox program.

**Themes**	**Contents**	**Session**
Introduction	What is depression?	Session 1
	What is MoodBox?	Session 1
	Introduction of the process of MoodBox	Session 1
	Symptom assessment	Session 1
Cognitive intervention	Learning automatic thoughts	Sessions 2–6
	Identify distortion thoughts	Sessions 2–6
	Practicing cognitive intervention techniques	Sessions 7–10
	Review and testing	Session 11
Interpersonal intervention	Interpersonal problem diagnoses	Session 12
	Learning interpersonal theory	Session 13
	Practicing interpersonal intervention	Sessions 14–17
	Review and testing	Session 18
Relaxation	Mindfulness meditation	Sessions 2–10 and Sessions 12–17
	Relaxation music	Sessions 2–6
Review and termination		Sessions 19–20

#### Online Psychoeducation Group

For the online psychoeducation group, a psychoeducation website will be provided. The website will deliver psychoeducation that includes general information about depression and tips for managing depressive moods. However, the psychoeducation website does not contain any therapeutic contents. Participants will be asked to visit the website twice a week during the 8-week intervention period and will be followed up continuously for 1 year.

#### Naturalistic Observation Group

Participants in the observation group will only receive regular follow-up at the same visit time point with the MoodBox group and will be followed up continuously for 1 year.

### Outcome Measures and Data Collection Procedure

The follow-up procedure has been summarized in Table 2, including the outcome measures and the assessment time points of the study. Other information includes demographics information, medical history, and current treatment plan, which will be collected by questionnaires via WeChat public account. The adverse events will be assessed by participants' self-report and documented by the research team.

**Table 2:** Measures and visit time point of outcome assessment.

**Visit time point**	**Visit 1 (−7 to 0 days)**	**Visit 2 (day 0)**	**Visit 3 (14 ± 2 days)**	**Visit 4 (28 ± 2 days)**	**Visit 5 (42 ± 2 days)**	**Visit 6 (56 ± 7 days)**	**Visit 7 (84 ± 7 days)**	**Visit 8 (6 months ± 14 days)**	**Visit 9 (9 months ± 14 days)**	**Visit 10 (12 months ± 14 days)**
Informed consent	×									
Patient general demographics	×									
Medical history	×									
Physical and nervous system examination	×									×
DSM-IV diagnosis	×									×
Inclusion and exclusion criteria	×	×								
General information collection form		×	×	×	×	×	×	×	×	×
PHQ-9	×	×	×	×	×	×	×	×	×	×
GAD-7		×	×	×	×	×	×	×	×	×
ASRM		×	×	×	×	×	×	×	×	×
LES		×	×	×	×	×	×	×	×	×
CTQ		×								
IPPA		×								
EMBU		×								
CSQ		×								
Treatment status		×	×	×	×	×	×	×	×	×
Adverse events	×	×	×	×	×	×	×	×	×	×
Adherence to intervention			×	×	×	×				
Satisfaction and acceptability for the intervention			×	×	×	×				

*DSM-IV, Diagnostic and Statistical Manual of Mental Disorders (Fourth Edition); PHQ-9, Patient Health Questionnaire; GAD-7, Generalized Anxiety Disorder-7; ASRM, Altman Self-Rating Mania Scale; LES, Life Event Scale; CTQ, Childhood Trauma Questionnaire; IPPA, Inventory of Parent and Peer Attachment; CSQ, Coping Style Questionnaire*.

#### Primary Outcome

The primary outcome is the efficacy of the intervention measured by the PHQ-9 (22). PHQ-9 is a nine-item reliable and valid self-report depression severity screening and diagnostic tool based on the DSM-IV criteria (23, 24). Response options are “not at all,” “several days,” “more than half the days,” and “nearly every day,” scored as 0, 1, 2, and 3, respectively (total score range from 0 to 27). In the current study, efficacy is defined as the participants score ≥ 2 on less than two items on the PHQ-9 scale at post-treatment assessment. PHQ-9 has demonstrated high reliability and validity in the Chinese population, with Cronbach's alpha for the internal consistency reliability being 0.86, the correlation coefficient for the 2-week test–retest of the total score being 0.86, and positive correlation with the Self-Rating Depression Scale (SDS; *r* = 0.29, *p* < 0.001) and the 36-item Short Form Health Survey (SF-36; correlation coefficients ranged from −0.11 to −0.47, *p* < 0.001) (25).

#### Secondary Outcomes

##### Onset of Mood Events

The incidence of mood events of participants in each group (MoodBox group, online psychoeducation group, and naturalistic observation group) during the follow-up period will be assessed by (a) depressive episode: meet the diagnosis of a depressive episode as measured by PHQ-9 diagnostic algorithm or according to the DSM-IV diagnostic criteria; (b) hypomania or manic episode: the Altman Self-Rating Mania Scale (ASRM) scale total score ≥ 5 or meet the diagnosis of hypomania or manic episode based on DSM-IV diagnostic criteria.

ASRM is a five-item mania self-rating scale for assessing the severity of mania or hypomania (26). A cutoff score of 5 or higher indicates a high probability of a manic or hypomanic condition (based on a sensitivity rating of 85.5% and a specificity rating of 87.3%).

##### Response Rate

The response rate is defined as the proportion of participants who do not meet the criteria of subclinical depression of each study group during the follow-up period. The efficacy of the continuous intervention is defined as the participants showed a decrease of depression symptoms measured by PHQ-9 (less than two items of the scale score ≥ 2 and the total score <5) for all the post-treatment follow-up assessments after completing 8 weeks' intervention.

##### Participant Adherence to the Intervention

The adherence of participants to the assigned intervention will be measured by the usage of the intervention website. Specifically, we will measure the frequency of log-in, duration of website use, and completeness of homework.

##### Participant Satisfaction and Acceptability for the Intervention

The satisfaction and acceptability of the intervention will be assessed by questionnaires at the end of the intervention. In the questionnaire, the researchers asked about “how easy is it for you to understand the content?,” “how easy is it for you to use the program?,” “do you feel the program can help you manage your mental difficulties?,” “do you want to use the program in the future?,” and “will you recommend the program to other people?.” The participants will be asked to answer each question in a scale of 1–5. Qualitative interviews will also be conducted with participants who are willing to participate.

##### Contributing Factors of Subclinical Depression

In order to identify the contributing factors of subclinical depression, psychosocial outcomes including anxiety, life events, attachment style, coping style, and parenting style will also be assessed, which will be measured by the Generalized Anxiety Scale (GAD)-7, the Life Event Scale (LES), Childhood Trauma Questionnaire (CTQ), the Inventory of Parent and Peer Attachment (IPPA), Egna Minnen Beträffande Uppfostran (“own memories of parental rearing practices in childhood,” EMBU), and Coping Style Questionnaire (CSQ), relatively. The Generalized Anxiety Disorder-7 (GAD-7) is a seven-item self-report scale for assessing the severity of anxiety. Each item is scored from 0 to 3, with a total score of 21 (27). The Chinese version of the scale established good reliability (Cronbach's α = 0.90) (28). Negative life events will be measured by LES, a 48-item self-report scale to measure positive and negative life events (29). The scale measures events regarding serious illness, housing, relationships and social difficulties, relationship breakdowns, unemployment, and financial crisis. Childhood traumatic experiences will be assessed by CTQ (30). The CTQ consists of 28 items, with each item scored from 1 to 5 with a total score of 125. Attachment will be measured using IPPA, a 25-item scale consisting of two core components: parent attachments and peer attachments (31). The EMBU is used to measure perceived parents' raring behaviors (32), with a questionnaire comprising 81 questions and 15 subscales covering raring behaviors such as overinvolvement, affection, overprotectiveness, guilt engendering, and rejection. Coping strategies will be assessed using CSQ (33). The CSQ consists of 20 items to assess different ways of coping, with each item having four response options: “never used,” “occasionally used,” “sometimes used,” and “often used.”

### Statistical Consideration

Data management and statistical analysis will be conducted using SAS9.4 (SAS Institute Inc., Cary, NC, USA). The sample characteristics would be compared using chi-square test for categorical variables or using Fisher's Z test when needed; ANOVA tests for normal distributed variables; and Mann–Whitney's U test for non-normal distributed variables. Analysis of efficacy will be based on an intent-to-treat principle, comprising all participants randomized regardless of the treatment group. The Kaplan–Meier survival analysis will be used to calculate the estimated time from baseline to response during 8 weeks' follow-up.

The Cox proportional hazard regression model using the Breslow method will be used to compare estimated time to response between groups, with control for covariates such as age, gender, years of education, and disease-related conditions; also we would use the exact method as sensitive analysis in case of tied survival data. Considering the long interval between visits after week 8, we will also use maximum likelihood estimation for Cox hazard regression model following Shen's work (34) to control left interval truncated data. Secondary analyses will be performed to assess changes from baseline to the endpoint of the study on scores of scales, using mixed-effects model for repeated-measures analysis (MMRM). A *p*-value <0.05 is considered as statistically significant. The Bonferroni method will be used to control multiple comparisons of type I error inflation.

## Discussion

Although recommendations have been made to manage subclinical depression (35, 36), most people with subclinical depression do not receive any treatment (14–16), even though the risk of subclinical depression turning into a full-blown depressive disorder is high. In recent years, in China, an increasing number of mental health professionals recommend people with subclinical depression to receive psychological interventions. However, because the number of qualified psychotherapists is limited, there is a significant lack of accessibility for people with subclinical depression in China (16). Internet-based interventions are suitable for resource-limited settings with constrained mental health services and have the potential to be scaled up, which in turn will be a solution for scalable subclinical intervention.

MoodBox combined different evidence-based psychological interventions that are established as being effective to reduce depressive symptoms, including CBT, IPT, and mindfulness meditation. The theoretical background of existing internet-based psychological interventions is primarily based on CBT. By combining multiple evidence-based interventions, MoodBox offers a more comprehensive intervention and provides more treatment options. Due to the diverse symptom presentation of people with subclinical depression, a comprehensive intervention has the potential to cover a broader range of symptoms, which in turn may have a positive impact on treatment adherence, satisfaction, and acceptability. Second, we measured mood events to provide a comprehensive understanding of the reduction of subclinical depression on preventing mood disorders. Previous research mainly focused on the efficacy of preventing depression, without paying attention to the outcome of other mental health problems. Subclinical depression is not only an early sign of major depression but also an early sign of other mental health problems, such as bipolar disorder. There is a necessity to understand how the reduction of subclinical depression can contribute to the prevention of other mood events. Another strength of the study is the use of WeChat to send out notifications to participants for follow-up information, and the implementation of the electronic data capture (EDC) system for data management. WeChat is a messaging and social media app that has been wieldy used in China, especially for adolescents. There are over one billion active monthly users of WeChat. Therefore, sending follow-up notification using WeChat is an efficient method to manage follow-up visit remotely. Additionally, participants can complete scales on their phone using the link sent via WeChat, which will allow the data directly to enter the EDC database and mitigate any error that may be caused by entering data manually. This new follow-up method can improve the quality of the trial by enacting higher requirements for quality control. Additional, ethical issues and protecting subjects' privacy are also important during the follow-up by WeChat platform, as all the research staff will sign a confidentiality agreement and operate in strict compliance with the program provisions and ethical requirements.

There are some limitations of the study. First, all participants will be recruited from first-year university students in Beijing; thus, the sample might not be representative of the subclinical depression population in China. However, in light of the findings that the onset age of depression is during teenage years (37), adolescents and young adults are a high-risk population for subclinical depression. Moreover, university students from the three universities were recruited across the whole country; therefore, it is representative to a certain extent. Second, although the length of treatment and follow-up period (i.e., after the 8-week intervention, participants will be followed up for 1 year) is sufficient to demonstrate treatment effect, due to the relatively short follow-up period, the long-term effect of the intervention cannot be evaluated in this study.

To our knowledge, this is the first study to innovatively develop and test an intervention to improve psychological well-being and decrease the incidence of MDD in subclinical depression population. Once proven effective and acceptable, MoodBox could be potentially integrated into the routine clinical service to facilitate the management for people with subclinical depression.

## Ethics Statement

The study will be performed according to the ethical standards stated in the Declaration of Helsinki and its subsequent updates. All participants will be provided with an overview of the study's aims and characteristics summarized in the Informed Consent Form (ICF) and will be obtained written informed consent. The voluntary character of the study will be specified, indicating that withdrawal from the study is permitted at any time, without interfering with the usual treatment. The Ethics Committee Board of Beijing Anding Hospital approved the study protocol on 4 January 2020 (No. 202006FS-2).

## Author Contributions

GW secured grant funding and supervised the study design. XC designed the study and drafted the manuscript. XZha and XZhu reviewed and edited the manuscript. All authors contributed to the article and approved the submitted version.

## Conflict of Interest

The authors declare that the research was conducted in the absence of any commercial or financial relationships that could be construed as a potential conflict of interest.
